# P-1503. Estimating the Size of the Population at Risk of Pneumococcal Disease in the Unified Health System in Brazil from the Use of Outpatient Administrative Data

**DOI:** 10.1093/ofid/ofaf695.1687

**Published:** 2026-01-11

**Authors:** Ricardo Macarini Ferreira, Daniela V Pachito, Paulo Almeida, Rodrigo Alexandre

**Affiliations:** Pfizer Brazil, São Caetano do Sul, Sao Paulo, Brazil; Pfizer Brazil, São Caetano do Sul, Sao Paulo, Brazil; Pfizer Brazil, São Caetano do Sul, Sao Paulo, Brazil; Pfizer Brazil, São Caetano do Sul, Sao Paulo, Brazil

## Abstract

**Background:**

The guidance on the vaccination schedule against pneumococcal disease (PD) in Brazil is carried out by the National Immunization Program (NIP) in the Unified Health System (SUS) in Brazil., through the immunization recommendations presented by the Reference Centers for Special Immunobiologicals (Centros de Referência para Imunobiológicos Especiais, CRIE). Currently, CRIE guidelines specify two types of population: the high-risk population, for which immunization with the pneumococcal conjugate vaccine 13, with at least two booster doses of the polysaccharide vaccine 23 (PPS-23), is indicated, and the at-risk population, currently receiving the complete vaccination schedule composed solely of PPS-23 doses. Our objective was to estimate the population size living with health conditions assigned as ‘at risk’ for PD by analyzing administrative data in Brazilian Public Health System (SUS).

**Box 1:**
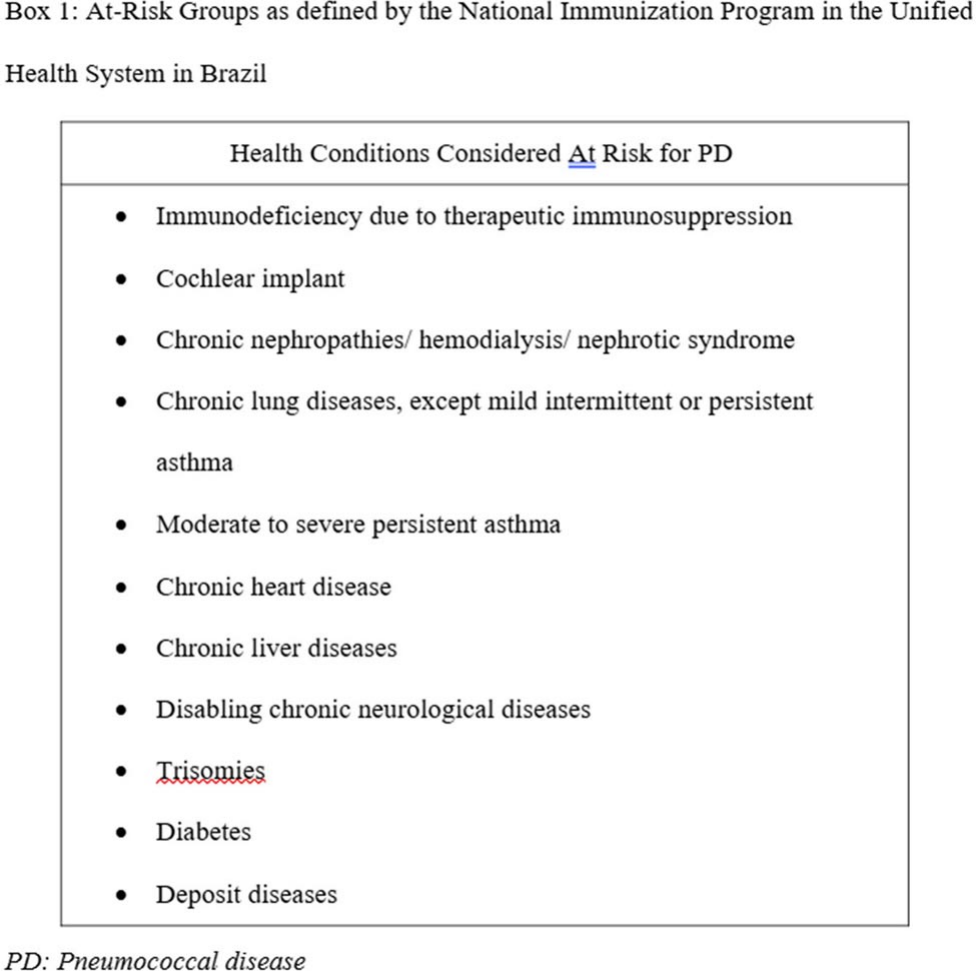
At-Risk Groups as defined by the National Immunization Program in the Unified Health System in Brazil

**Figure 1:**
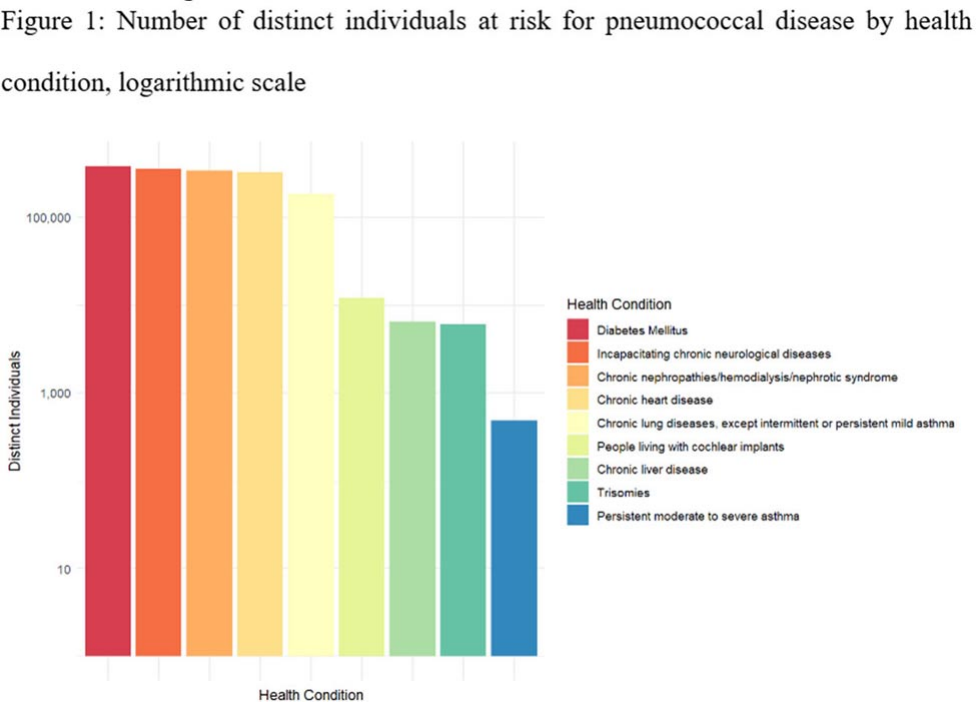
Number of distinct individuals at risk for pneumococcal disease by health condition, logarithmic scale

**Methods:**

Retrospective analysis of the outpatient information system (Sistema de Informação Ambulatorial, DATASUS-SIA). Health conditions considered to be at risk are presented in Box 1. ICD-10 and procedure codes related to these health conditions and proxies of disease activity and severity were created. Encrypted identifiers in DATASUS-SIA were used to allow for the identification and counting of distinct individuals, to avoid double counting (i.e., same individuals being accounted for in different categories), from November 2021 to October 2023.

**Results:**

A total of 1,608,659 distinct individuals at risk for PD were identified. Five categories accounted for most cases (98.5%), namely diabetes mellitus; incapacitating chronic neurological diseases; chronic nephropathies; chronic heart disease; and chronic lung diseases. (Figure 1). The lowest number of identified cases was for the group persistent moderate to severe asthma, for which the initial number of distinct identified individuals was 555,103 but only 491 (8.9%) of cases fulfilled proxies for disease activity and severity.

**Conclusion:**

To the best of our knowledge, this is the first study to provide estimates of the at-risk population size for PD in the SUS in Brazil. Such estimate is crucial to allow for future assessment of vaccination coverage rates and to underpin the design of new immunization programs.

**Disclosures:**

Ricardo Macarini Ferreira, Md/ MBA, Pfizer Brazil: Employee Daniela V. Pachito, MD PhD MBA, Pfizer: Employee|Pfizer: Stocks/Bonds (Private Company) Paulo Almeida, PhD, Pfizer Brazil: Employee

